# Spectroscopic Studies of Lanthanide(III) Complexes with L-Malic Acid in Binary Systems

**DOI:** 10.3390/ijms25179210

**Published:** 2024-08-25

**Authors:** Michał Zabiszak, Justyna Frymark, Jakub Grajewski, Renata Jastrzab

**Affiliations:** Faculty of Chemistry, Adam Mickiewicz University in Poznan, Uniwersytetu Poznanskiego 8, 61-614 Poznan, Poland; justyna.frymark@amu.edu.pl (J.F.); jakub.grajewski@amu.edu.pl (J.G.); renatad@amu.edu.pl (R.J.)

**Keywords:** lanthanide(III) complexes, malic acid, spectroscopy, potentiometric measurements

## Abstract

Binary systems of lanthanide ions (La, Nd, Gd, Ho, Tb, and Lu) with L-malic acid in molar ratios of 1:1 and 1:2 were studied. This study was carried out in aqueous solutions, and the composition of the formed complexes was confirmed using computer data analysis. The overall stability constants of the complexes and the equilibrium constants of the reaction were determined. The effect of ligand concentration on the composition of the internal coordination sphere of the central atom was observed. Changes in the coordination sphere of lanthanide ions were confirmed by spectroscopic measurements.

## 1. Introduction

Lanthanides have become a topic of significant interest in recent years due to their potential applications and unique properties, sparking a growing interest in the use of their compounds in various fields [1,2,3,4,5]. These elements are indispensable in industry, playing an essential role in applications ranging from catalysis and petroleum refining to the production of powerful magnets used in electronics and renewable energy technologies [6,7,8,9]. Their distinctive properties contribute to advancements in manufacturing, telecommunications, and green energy, underscoring their critical role in modern industrial processes [10,11,12,13]. In addition to their industrial applications, lanthanides are increasingly recognised for their potential within biological systems, due to their catalytic, magnetic, and luminescent properties when combined with organic ligands [10,14,15,16,17]. These applications extend to medicine, where lanthanides have been used as contrast agents for magnetic resonance imaging and as radiotherapeutic drugs [18,19]. Certain lanthanide ions show promise in influencing cellular functions, particularly in the context of cancer research [20,21,22]. However, addressing concerns about lanthanide toxicity is crucial to realising its full medical potential [23,24]. Therefore, it is imperative to elucidate the interactions of lanthanides with bioligands that occur within living organisms. This knowledge may contribute to the development of safe and effective lanthanide-based therapeutics and diagnostic tools.

Malic acid, an organic alpha-hydroxy acid that occurs naturally in fruits and living organisms, plays a crucial role in various biochemical processes [25,26,27]. It possesses two carboxyl groups and a hydroxyl group, and while it exists in two different stereoisomeric forms, only the L-isomer is found in nature. Its significance lies in its involvement in biochemical processes such as the Krebs cycle, which is essential for the production of cellular energy, and its role as a ‘building block’ for chemical synthesis [28,29]. In addition to its biochemical functions, malic acid finds diverse applications, serving as an acidic agent to enhance flavours [30]. It is also used as a pH regulator and exfoliating agent in cosmetics to remove dead skin cells [31,32]. Furthermore, its compatibility with specific drug formulations makes it valuable in the industry, and its ability to enhance solubility and stability further expands its utility [33,34,35]. Moreover, because of its ability to form stable complexes by coordinating with metal ions through carboxyl and hydroxyl groups, malic acid is proven to be an effective complexing agent.

The intriguing properties and potential applications of lanthanide ions have motivated us to continue our systematic investigations into their interactions with various molecules [36,37,38]. Our recent research has primarily focused on examining the formation of complexes in the binary system of L-malic acid with lanthanide ions. The results of this investigation could significantly enhance our understanding of the coordination chemistry between lanthanide ions and malic acid, providing insight into potential applications for lanthanide complexes in catalysis, medicine, and materials science. This article presents the findings from potentiometric and spectroscopic studies on complex formation in systems involving lanthanide and lanthanide ions.

## 2. Results and Discussion

### 2.1. Equilibrium Study

Data from the previous literature show the possibility of forming complex compounds of lanthanide ions with malic acid [39,40,41]. Previous studies have been conducted under other measurement conditions, including temperature, ionic strength, electrolyte, inert gas atmosphere, and pH range. Other complex forms were also found, and analogous forms showed similar values of stability constants. Noteworthy is the fact that different methods of obtaining complexes were used, their stability constants were not shown, and their form distribution diagrams were not shown.

In the binary systems studied, the composition and overall stability constants (log*β*) of the complexes formed were determined by potentiometric measurements (Table 1). The protonation constants of malic acid and the hydrolysis constant of lanthanide(III) ions served as inputs for theoretical calculations [36,37]. Notably, we observed similarities in the formation of analogous complex forms compared to those in our previously published studies on the complexation of O-donor ligands with lanthanide ions. In tartaric acid systems, a different coordination behaviour was observed compared to the lanthanides studied, particularly in their interaction with lanthanum(III) ions [36]. In addition, consistent complex compositions for neodymium(III), gadolinium(III), terbium(III), holmium(III), and lutetium(III) ions were identified within the tested pH range, reflecting those formed by europium(III) ions [38]. The divergence in our results from those published for similar systems can be attributed to differences in measurement conditions and the spectroscopic methods employed to analyse the internal coordination sphere. Furthermore, included in Table 1 are the protonation constants for malic acid that were published in our earlier work [37].

**Figure 1:**
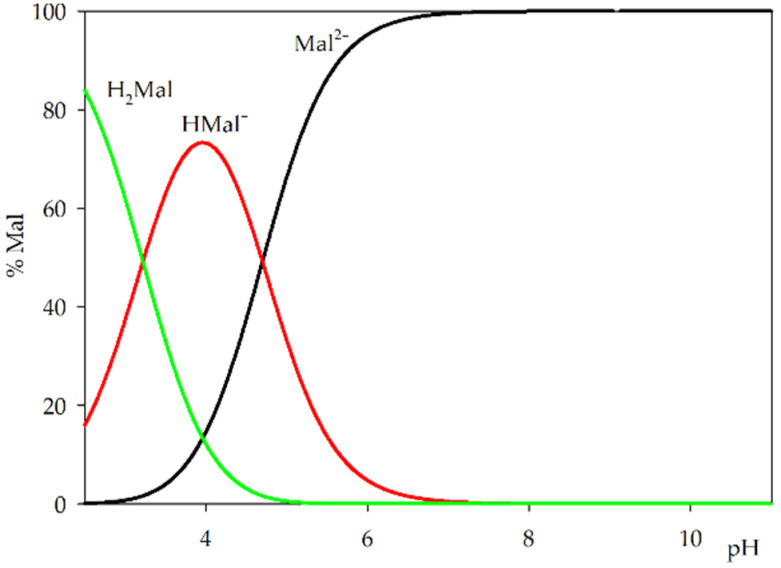
Distribution diagram for L-malic acid.

The distribution diagram for protonated forms of L-malic acid indicates that even at low pH values, carboxyl groups can form coordination bonds with the central atom (Figure 1). No deprotonation of the hydroxyl group was observed in the pH range studied; however, the presence of metal ions may contribute to its earlier deprotonation and presence in the internal coordination sphere at higher pH values.

**Figure 2:**
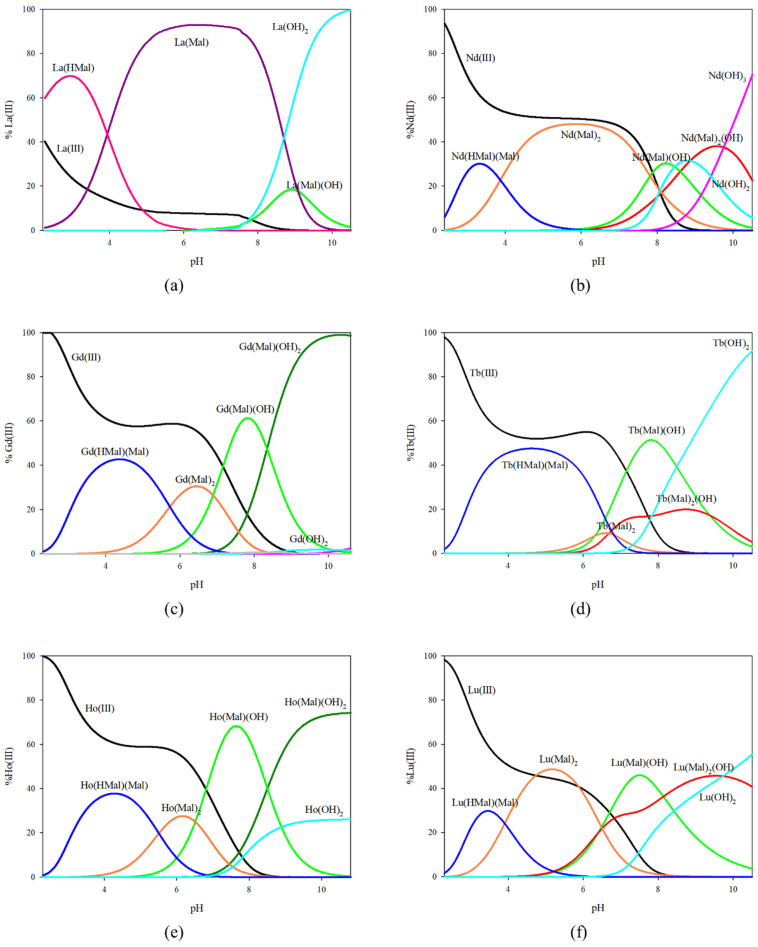
Distribution diagram for the equimolar systems studied: (**a**) La(III)/L-malic acid; (**b**) Nd(III)/L-malic acid; (**c**) Gd(III)/L-malic acid; (**d**) Tb(III)/L-malic acid; (**e**) Ho(III)/L-malic acid; (**f**) and Lu(III)/L-malic acid.

**Figure 3:**
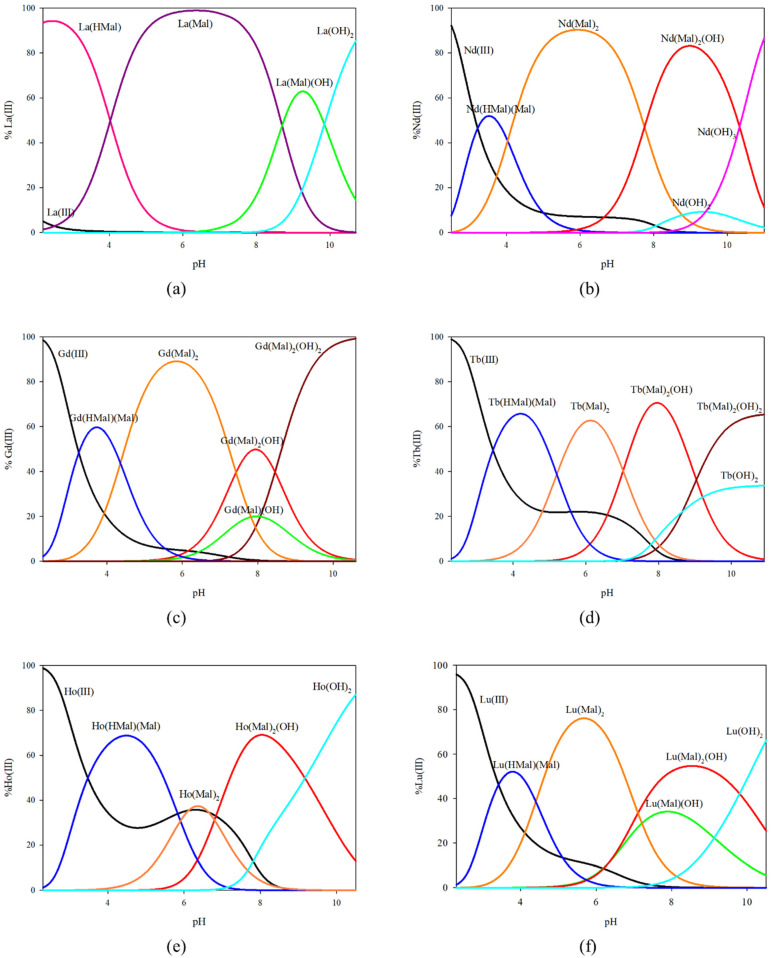
Distribution diagram for the systems studied with excess of L-malic acid: (**a**) La(III)/L-malic acid; (**b**) Nd(III)/L-malic acid; (**c**) Gd(III)/L-malic acid; (**d**) Tb(III)/L-malic acid; (**e**) Ho(III)/L-malic acid; and (**f**) Lu(III)/L-malic acid.

On the basis of the values of stability constants obtained, graphs of the distribution of complex forms in equimolar systems (Figure 2) and with an excess of malic acid (Figure 3) were prepared. In the equimolar system, as in the systems with tartaric acid, the formation of complex forms with only one acid molecule in the inner coordination sphere was observed for lanthanum ions. The La(HMal) complex is already present in the system below the tested pH range, and at the maximum concentration, 70% of the metal ions participate in coordination. The dominant form of lanthanum ions in the pH range of 4.0–9.0 is the La(Mal) complex, where about 90% are bound by malic acid molecules. In addition, at high pH values, a hydroxycomplex of La(Mal)(OH) is observed, but it is not the dominant form. For the other metal ions studied, the formation of a different set of complex forms was observed, which is reflected in the distribution diagrams. At pH 2.5, the first observed complex form is formed whose inner coordination sphere of the metal ion contains a partially deprotonated form of malic acid and a fully deprotonated form of its molecule. As the pH increased, the M(Mal)_2_ form was found to bind between 10% (Tb^3+^) and 50% of lanthanide ions (Lu^3+^). In addition, for all equimolar systems, the hydroxyl form of Ln(Mal)(OH) is observed, which is the dominant form for all lanthanide ions, excluding lanthanum ions. It should be noted that for the Gd(III) and Ho(III) ions at basic pH values, the dominant form is the Ln(Mal)(OH)_2_ compound, while for the Nd(III), Tb(III), and Lu(III) ions under the same pH conditions there is a complex of the type Ln(Mal)_2_(OH).

For the systems studied with an excess of malic acid, the formation of a different set of complex forms was observed compared to that observed for the equimolar systems for neodymium, gadolinium, terbium, and holmium ions. The same complex forms were observed for lanthanum and lutetium ions. The excess amount of malic acid results in an increased contribution of lanthanum ions to the formation of complex forms, which is most noticeable for the La(Mal)(OH) complex, which becomes the dominant complex at pH around 9.0 binding nearly 65% of the metal ions. On the other hand, for lutetium ions, the monohydroxyl form in excess malic acid ceases to be the dominant form in favour of the Lu(Mal)_2_(OH) complex. The absence of the M(Mal)(OH) form is observed for the neodymium, terbium, and holmium ion systems, and the M(Mal)(OH)_2_ form for the gadolinium ion system. The system containing neodymium and holmium ions with an excess amount of ligand contains only complex forms in which there are two molecules of malic acid in the internal coordination sphere, where they all dominate and bind 40% to 90% of neodymium ions. Malic acid in double excess with gadolinium ions forms Gd(HMal)(Mal), Gd(Mal)_2_, Gd(Mal)_2_(OH), Gd(Mal)_2_(OH)_2_, and Gd(Mal)(OH), where the monohydroxyl form containing one molecule of malic acid is not the dominant form. In terms of the gadolinium ions, four complex forms were found having in their structure two molecules of the ligand under study. Compared to the equimolar system containing the same metal ions, the complexation process is more effective, resulting in the dominance of these complex forms, which was not observed when the system was studied at a molar ratio of 1:1.

**Figure 4:**
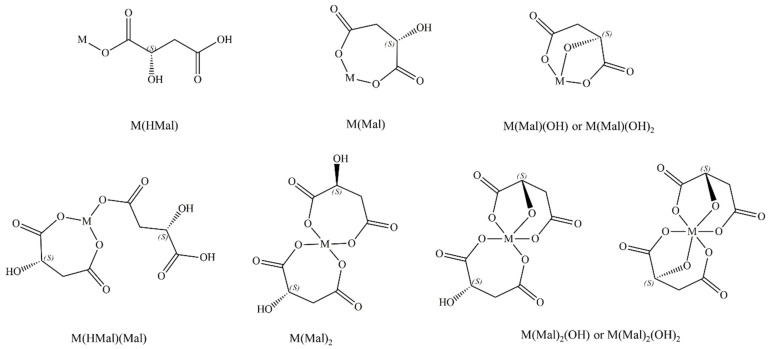
Possible configurations of L-malic acid with lanthanide ions in formed complexes.

On the basis of the data obtained from thermodynamic studies, the composition of the complexes formed in the studied systems was determined; a method of coordinating malic acid with the central atom was proposed (Figure 4). The protonation constants for malic acid indicate that mainly carboxyl groups are involved in coordination, and the hydroxyl group can occur in the internal coordination group at high pH values. The deprotonation of the hydroxyl group caused by the presence of metal ions in the system may participate in coordination during the formation of hydroxycomplexes.

### 2.2. Luminescence Spectroscopy

**Figure 5:**
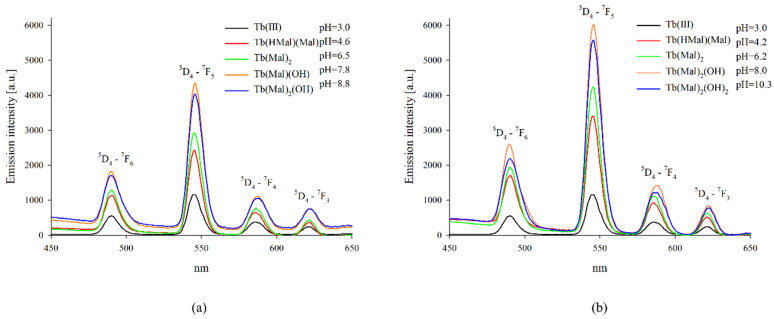
Emission spectra of the systems: (**a**) Tb(III)/L-malic acid (1:1 ratio); (**b**) Tb(III)/L-malic acid (1:2 ratio).

On the basis of the obtained distribution diagrams, luminescence measurements were made for binary systems containing terbium(III) ions (Figure 5). Measurements were made at pH values at which the highest contribution of metal ions to the formation of the complex form was found. No emission was observed from the solvent or from the free malic acid. The maximums of Tb(III) emission in complexes with malic acid were found to appear at wavelengths similar to those of the respective uncomplexed metal ions. As the pH value increased and the coordination environment of the central atom changed, changes in emission intensities were observed that corresponded to the transitions from the ^5^D_4_ level to ^7^F_j_ (j = 6, 5, 4, 3). The most intensive emissions were observed in the region corresponding to ^5^D_4_-^7^F_5_, and medium-strong emissions in the regions of rest transitions of the Tb(III) ion. For the system with a double excess of malic acid, where a higher proportion of metal ions was found to be involved in the coordination process, a higher emission intensity was observed compared to that of the equimolar system. The observed changes in emission intensity compared to those of free metal ions indicate the substitution of water molecules in the internal coordination sphere of terbium ions. In addition, increasing the concentration of the ligand affects the coordination environment of the central atom.

### 2.3. IR Spectroscopy

**Figure 6:**
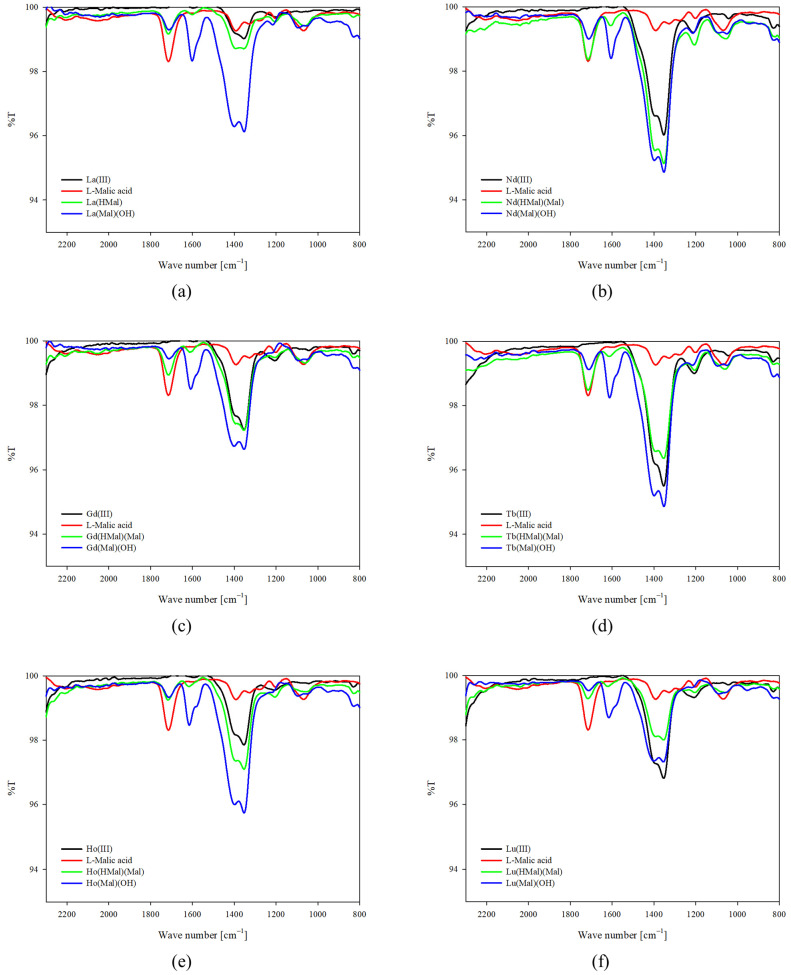
IR spectra for the equimolar systems studied: (**a**) La(III)/L-malic acid; (**b**) Nd(III)/L-malic acid; (**c**) Gd(III)/L-malic acid; (**d**) Tb(III)/L-malic acid; (**e**) Ho(III)/L-malic acid; and (**f**) Lu(III)/L-malic acid.

**Figure 7:**
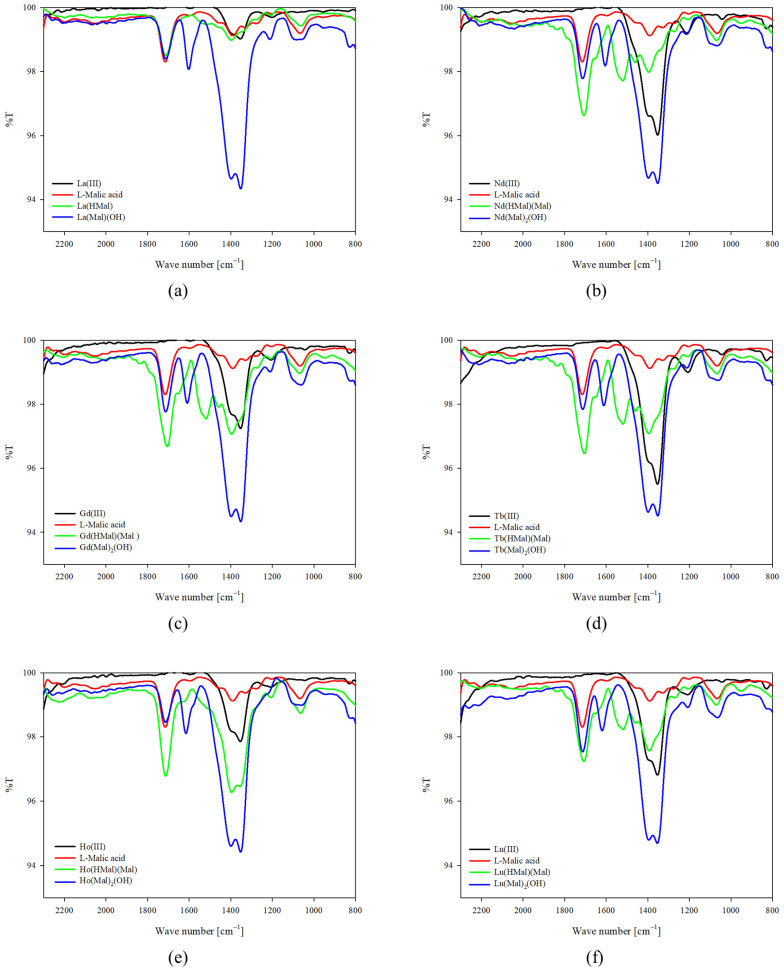
IR spectra for the systems studied with excess of L-malic acid: (**a**) La(III)/L-malic acid; (**b**) Nd(III)/L-malic acid; (**c**) Gd(III)/L-malic acid; (**d**) Tb(III)/L-malic acid; (**e**) Ho(III)/L-malic acid; and (**f**) Lu(III)/L-malic acid.

The contribution of malic acid functional groups to the coordination was confirmed using infrared (IR) spectroscopy by comparing the spectra of the ligand itself with those of the complexes obtained in the studied systems. Figures show the obtained spectra of protonated complexes and hydroxycomplexes (Figure 6 and Figure 7). Similarly to luminescence studies, spectra were recorded at the pH dominance of complex compounds. In equimolar systems, it was observed that the intensity of the characteristic bands of the carboxyl groups of the malic acid molecule (1720 cm^−1^) decreases with increasing pH. It was observed that in systems with an excess of alpha-hydroxyacid, for which two of its molecules were found in the internal coordination sphere of the metal-ion coordination sphere, the intensity of the bands corresponding to the stretching vibrations of the C=O bond increases with a decrease in the concentration of hydrogen ions in the studied system. These differences may be due to the greater participation of lanthanide ions in the complexation process with increased ligand concentration. Additionally, in the range of symmetric stretching vibrations of C-O bonds (ν_asC-O_ 1450–1350 cm^−1^), both band shifts and a change in their intensity are observed compared to the spectra of free metals and the ligand, suggesting the formation of coordination bonds between the central atom and the other functional groups of the malic acid molecule. It is noteworthy that for all hydroxycomplexes, the appearance of a band was observed at 1610 cm^−1^, which is attributed to vibrations of deformation in compounds containing a hydroxyl group, confirming the participation of this group in complexation.

### 2.4. CD Spectroscopy

The CD and corresponding UV spectra were recorded for all Ln systems with 1:1 and 1:2 molar ratios with L-malic acid in the range of 185–400 nm at a concentration of 0.001 M. This concentration allows for a direct comparison with the results of the titration measurements. These measurements at relatively high concentrations were possible by using a sample cell with a 0.5 mm optical path. Both series of complexes were measured at a pH determined on the basis of the results obtained from computer analysis of potentiometric data.

As L-malic acid did not have aromatic substituents or other commonly used chromophores to analyse conformational structures, the analysis of the conformation of the carbon chain, and thus the interactions of the acid with Ln ions, was carried out based on the observation of Cotton effects derived from the n-π * electron transitions of carboxylic groups in this molecule. Therefore, the pictures of the CD spectra are limited to the range between 185 and 350 nm.

It is known that in both protonated and deprotonated forms, L-malic acid mainly adopts the trans conformation of its four-carbon backbone, but because of the ionisation and rearrangement of intramolecular hydrogen bonds, its CD spectra change at different pH. L-malic acid in aqueous solutions exhibits a single positive Cotton effect = 1.3 around 211 nm, and its simple divalent salts (Na, K, and Li) show a positive Cotton effect = 3.8 at 208 nm. These changes must be taken into consideration during the analysis of Ln complexes to understand but not overestimate the role of the central ion.

Studies on the conformations of malic acid complexes were divided into two parts, for equimolar systems and for systems with an excess of malic acid. In both cases, it should be taken into account that the form present at a given pH is not the only one in the solution and that the CD spectrum shows the populations of all conformers present in the solution at a given pH.

The overall pattern of Cotton effects observed for all 1:1 systems measured shows that in acidic solutions of Ln complexes of malic acid chelate complexes, the following are present: In such structures, the carbon skeleton of L-malic acid bends to the gauche conformation, and this is associated with the occurrence of negative Cotton effects around 212 nm on the CD spectra [43,44]. On the basis of potentiometric measurements, a smaller absolute value of the Cotton effect was observed for La complexes, which may be due to the fact that La coordinates only one acid molecule. In the neutral and basic pH, in turn, a population of trans conformers of L-malic acid starts to be present in solutions, which can be caused by electrostatic repulsions of negatively charged carboxylates and increased participation in coordination of the central ion by OH^−^ ligands. This significant share of the trans conformer in the mixture is visible in the spectra as a decrease in the negative Cotton effect [45].

**Figure 8:**
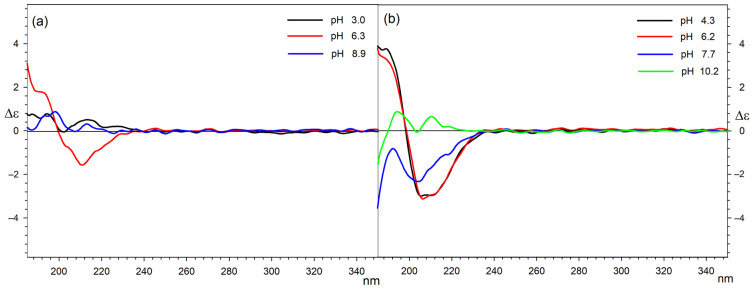
The CD spectra of the 1:1 systems: (**a**) La/Mal at pH 3.0, 6.3, and 8.9; (**b**) Ho/Mal at pH 4.3, 6.2, 7.7, and 10.2.

Figure 8 presents a comparison of the circular dichroism spectra that illustrate the complexation of lanthanum and Holm (as the representative of the other examined lanthanides) by L-malic acid at different pH levels.

For systems in which there is a two-fold excess of malic acid in relation to the central ion, dependencies are similar to those observed for equimolar mixtures. The greatest differences can be observed for the system with La as the central ion. In this case, the positive Cotton effect occurs at both acidic and alkaline pH. This can be explained by the participation of a single acid molecule in the coordination sphere of lanthanum. The second, uncoordinated molecule adopts a *trans* conformation in solution. According to the distribution diagram, in this system at a pH equal to 2.5, there is a complex with the partially protonated acid and, therefore, also partially present in the *trans* conformation. The clearly positive Cotton effect for the 1:2 system with lanthanum in an alkaline environment is the result of both the coordination of only one acid molecule and the participation of hydroxyl groups in the complexation of the central ion. The remaining ions in complexes with a stoichiometry of 1:2, due to the possibility of complexing two acid molecules, may therefore exist in a bent conformation [46]. They display negative values of Cotton effects for acidic solutions, which decrease with increasing pH. In the case of 1:2 Gd and Tb complexes, a positive Cotton effect can be observed for basic solutions, which is consistent with the distribution measurements because of the presence of the Gd(Mal)_2_(OH)_2_ and Tb(Mal)_2_(OH)_2_ complexes, respectively, in which hydroxyl groups involved in the coordination of the central ion displace acid molecules from the chelate complex.

**Figure 9:**
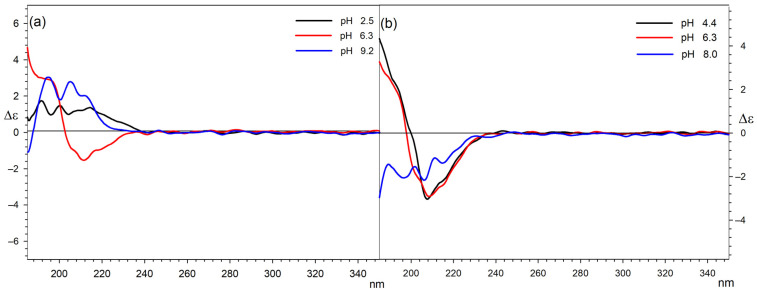
The CD spectra of the 1:2 systems: (**a**) La/Mal at pH 2.5, 6.3, and 9.2; (**b**) Ho/Mal at pH 4.4, 6.3, and 8.0.

A comparison of the circular dichroism spectra illustrating 1:2 (a) lanthanum and (b) Holm (as the representative of the other examined lanthanides) and (*S*)-malic acid at different pH values is presented in Figure 9.

The CD data for all 1:1 and 1:2 Ln:L-Malic acid systems are listed in Table 2 and Table 3, respectively. To preserve the clarity of the tables, the lines were compiled according to the increasing pH values.

In summary, it can be stated that the conformation of malic acid molecules in Ln complexes is very much dependent on the pH and, in the case of La ions, on the possibility of forming a complex with only one acid molecule. Regardless of the analysed system, the share of complexation by hydroxyl groups increases with increasing pH and this significantly increases the population of *trans* conformers of malic acid. The increase in *trans* conformers at high pH is also caused by an increase in the share of metal in the form of hydroxide, which is therefore not involved in the formation of complexes.

## 3. Materials and Methods

### 3.1. Materials

L-malic acid (Mal) was obtained from Merck and used without further purification. Lanthanide(III) nitrates (La(III), Nd(III), Gd(III), Tb(III), Ho(III), and Lu(III)) were obtained from Sigma-Aldrich (Darmstadt, Germany) and used without further purification. Lanthanide(III) nitrate solutions as well as α-hydroxy acids were prepared using demineralised carbonate-free water (conductivity 0.055 µS).

### 3.2. Equilibrium Study

The way in which the equipment was prepared and the potentiometric measurements were made, the conditions under which the measurements were carried out, and how the calculations were performed are described in detail in our previous works [36,37,38].

### 3.3. UV-Vis and Luminescence Spectroscopy

The samples were prepared in ultra-high-quality water using a Simplicity Ultrapure Water System (Millipore, Darmstadt, Germany). UV absorption spectra were recorded with a UV-1900 spectrophotometer (Shimadzu, Kyoto, Japan), and the concentration of metal ions was 0.001 M. The luminescence studies were recorded on a spectrofluorophotometer RF-600 (Shimadzu, Kyoto, Japan) using 5.0/5.0 nm slit widths. The concentration of terbium(III) ions was 0.001 M, and the complex samples were excited at 370 nm.

### 3.4. Infrared Spectroscopy (FT-IR)

Infrared spectra were recorded on an FT-IR INVENIO R spectrophotometer (Bruker, Bremen, Germany). Samples were prepared by dissolving L-malic acid and lanthanide(III) ions in D_2_O. The metal concentration for the IR studies was 0.1 M. The pH values were adjusted by the addition of NaOD or DCl. The pH values were corrected according to the formula pD = pH meter reading + 0.4 [47].

### 3.5. CD Spectroscopy

CD and UV spectra were recorded on the J810 spectropolarimeter (JASCO, Tokyo, Japan) at ambient temperature. Spectra were recorded in the range of 185–400 nm in water solutions at 200 nm/min, with a data pitch of 0.5 nm. The measurements were made in an N_2_ atmosphere (flow 15 L/min), and the optical path length was 0.5 mm. Due to the poor signal-to-noise ratio, 8 scans for L-malic acid complexes were accumulated, and the concentrations of the solutions were 1 × 10^−3^ mol dm^−3^, to keep the absorbance at an acceptable level.

## 4. Conclusions

Complexes formed by binary systems containing L-malic acid with selected lanthanide ions were studied spectroscopically. It was found that the systems with lanthanum ions form complex compounds containing only one ligand molecule in the internal coordination sphere, while for the other tested ions, the participation of two acid molecules in the coordination was confirmed. For this purpose, the sets of complex forms formed and the conditions of their dominance were determined on the basis of potentiometric studies. Furthermore, it was observed that the change in the concentration of L-malic acid affects the type of complex compounds formed in the systems studied. The formation of protonated complexes, simple complexes, and hydroxycomplexes was found. Luminescence studies have shown that, as the pH increases, an increase in the intensity of the emission corresponding to the energy transitions of the central atom is observed, which is evidence of a change in its coordination sphere. On the basis of infrared spectroscopy studies, the involvement of functional groups in the formation of coordination bonds with lanthanide ions was confirmed. When measurements were made under different pH conditions, changes in the internal coordination sphere caused by the attachment of successive functional groups to the central atom were confirmed. On the basis of circular dichroism studies, the conformational changes of L-malic acid in the complexes were confirmed. These studies confirmed the contribution of one acid molecule to coordination in systems with lanthanum ions compared to other lanthanides, as evidenced by the recorded magnitude of Cotton effects for the complexes studied. In addition, conformational changes were found due to the conditions of the measurements carried out, which is due to a change in the coordination sphere of the central atom, and the repulsion of negatively charged carboxylates and increased coordination of the hydroxyl group are observable.

## Figures and Tables

**Table 1:** The overall stability constants (log*β*) and the equilibrium constants of formation (log*K_e_*) of malic acid and the complexes formed in the binary systems of lanthanide(III) ions/L-malic acid (standard deviations are given in parentheses).

Species	log*β*	log*K_e_*	Reaction
**Mal**			
H_2_L	7.92(1) *	3.22	HL^−^ + H^+^ ⇋ H_2_L
HL	4.70(1) *	4.70	L^2−^ + H^+^ ⇋ HL^−^
**La(III)-Mal**			
LaHMal	9.52(5)	4.82	La^3+^ + HMal ⇄ La(HMal)
LaMal	5.54(3)	5.54	La^3+^ + Mal ⇄ LaMal
La(Mal)(OH)	−3.35(6)	4.88	LaMal + H_2_O ⇄ La(Mal)(OH)^2−^ + H^+^
**Nd(III)-Mal**			
Nd(HMal)(Mal)	13.19(6)	8.49	Nd ^3+^ + HMal^−^ + Mal^2−^ ⇄ Nd(HMal)(Mal)
Nd(Mal)_2_	9.24(6)	9.24	Nd ^3+^ + 2Mal^2−^ ⇄ Nd(Mal)_2_^−^
Nd(Mal)(OH) **	−3.03(7)	10.74	Nd^3+^ + Mal^2−^+ H_2_O ⇄ Nd(Mal)(OH) + H^+^
Nd(Mal)_2_(OH)	1.31(2)	5.84	Nd(Mal)_2_^−^ + H_2_O ⇄ Nd(Mal)_2_(OH)^2−^ + H^+^
**Gd(III)-Mal**			
Gd(HMal)(Mal)	12.61(3)	7.91	Gd^3+^ + HMal^−^ + Mal^2−^ ⇄ Gd(HMal)(Mal)
Gd(Mal)_2_	7.57(5)	7.57	Gd ^3+^ + 2Mal^2−^ ⇄ Gd(Mal)_2_^−^
Gd(Mal)(OH)	−3.26(5)	10.51	Gd^3+^ + Mal^2−^+ H_2_O ⇄ Gd(Mal)(OH) + H^+^
Gd(Mal)(OH)_2_ **	−11.83(4)	5.20	Gd(Mal)(OH) + H_2_O ⇄ Gd(Mal)(OH)_2_^−^ + H^+^
Gd(Mal)_2_(OH) ***	0.67(6)	6.87	Gd(Mal)_2_^−^ + H_2_O ⇄ Gd(Mal)_2_(OH)^2−^ + H^+^
Gd(Mal)_2_(OH)_2_ ***	−7.78(4)	5.32	Gd(Mal)(OH) + H_2_O ⇄ Gd(Mal)(OH)_2_^−^ + H^+^
**Tb(III)-Mal**			
Tb(HMal)(Mal)	12.81(3)	8.11	Tb^3+^ + HMal^−^ + Mal^2−^ ⇄ Tb(HMal)(Mal)
Tb(Mal)_2_	6.80(4)	6.80	Tb^3+^ + 2Mal^2−^ ⇄ Tb(Mal)_2_^−^
Tb(Mal)(OH) **	−3.46(4)	10.31	Tb^3+^ + Mal^2−^+ H_2_O ⇄ Tb(Mal)(OH) + H^+^
Tb(Mal)_2_(OH)	−0.06(1)	6.91	Tb(Mal)_2_^−^ + H_2_O ⇄ Tb(Mal)_2_(OH)^2−^ + H^+^
Tb(Mal)_2_(OH)_2_ ***	−8.98(9)	4.85	Tb(Mal)_2_(OH)^2−^ + H_2_O ⇄ Tb(Mal)_2_(OH)_2_^3−^ + H^+^
**Ho(III)-Mal**			
Ho(HMal)(Mal)	12.56(2)	7.86	Ho^3+^ + HMal^−^ + Mal^2−^ ⇄ Ho(HMal)(Mal)
Ho(Mal)_2_	6.86(6)	6.86	Ho^3+^ + 2Mal^2−^ ⇄ Ho(Mal)_2_^−^
Ho(Mal)(OH) **	−3.00(3)	10.77	Ho^3+^ + Mal^2−^+ H_2_O ⇄ Ho(Mal)(OH) + H^+^
Ho(Mal)(OH)_2_ **	−11.50(4)	5.27	Ho(Mal)(OH) + H_2_O ⇄ Ho(Mal)(OH)_2_^−^ + H^+^
Ho(Mal)_2_(OH) ***	−0.36(3)	6.55	Ho(Mal)_2_^−^ + H_2_O ⇄ Ho(Mal)_2_(OH)^2−^ + H^+^
**Lu(III)-Mal**			
Lu(HMal)(Mal)	12.70(2)	8.00	Lu^3+^ + HMal^−^ + Mal^2−^ ⇄ Lu(HMal)(Mal)
Lu(Mal)_2_	8.55(2)	8.55	Lu^3+^ + 2Mal^2−^ ⇄ Lu(Mal)_2_^−^
Lu(Mal)(OH)	−2.32(3)	11.45	Lu^3+^ + Mal^2−^+ H_2_O ⇄ Lu(Mal)(OH) + H^+^
Lu(Mal)_2_(OH)	1.74(4)	6.96	Lu(Mal)_2_^−^ + H_2_O ⇄ Lu(Mal)_2_(OH)^2−^ + H^+^

* The literature data of protonation constants of malic acid: log*β*_HL_ = 4.68 log*β*_H_2_L_ = 7.92 [42]; ** formed only in the equimolar system; *** formed only in a system with excess L-malic acid.

**Table 2:** Δε values for all 1:1 Ln(III)/Mal systems at different pH values in water solutions.

La(III)/Mal	Nd(III)/Mal	Gd(III)/Mal	Tb(III)/Mal	Ho(III)/Mal	Lu(III)/Mal
pH = 3.0 Δε (nm) 0.44 (213) 0.67 (195)	pH = 3.3 Δε (nm) −0.71 (215) −0.85 (208) 1.77 (191)	pH = 4.4 Δε (nm) −2.56 (207)	pH = 4.6 Δε (nm) −2.43 (208) 4.11 (186)	pH = 4.3 Δε (nm) −2.97 (210) −3.00 (206) −3.78 (189)	pH = 3.3 Δε (nm) −2.05 (210)
pH = 6.3 Δε (nm) −1.57 (211) 1.78 (192)	pH = 6.0 Δε (nm) −1.94 (212)	pH = 6.5 Δε (nm) −2.31 (207)		pH = 6.2 Δε (nm) −3.13 (207)	pH = 5.1 Δε (nm) −3.09 (210)
	pH = 8.2 Δε (nm) −0.75 (220) −0.79 (212) 0.78 (196)	pH = 7.7 Δε (nm) −1.73 (207) 0.61 (191)	pH = 7.8 Δε (nm) −1.23 (214) −1.69 (204) −1.26 (196)	pH = 7.7 Δε (nm) −1.10 (218) −2.33 (205)	pH = 7.5 Δε (nm) −2.45 (207) −2.41 (201)
pH = 8.9 Δε (nm) 0.32 (213) 0.88 (198)	pH = 9.5 Δε (nm) −0.19 (223) 0.44 (213) 0.86 (203) 1.14 (194)	pH = 9.2 Δε (nm) −0.45 (223) 0.52 (194)		pH = 10.2 Δε (nm) 0.67 (211) 0.89 (194)	pH = 9.3 Δε (nm) −0.54 (215) −0.93 (207) −1.08 (197)

**Table 3:** Δε values for all 1:2 Ln(III)/Mal systems at different pH values in water solutions.

La(III)/Mal	Nd(III)/Mal	Gd(III)/Mal	Tb(III)/Mal	Ho(III)/Mal	Lu(III)/Mal
pH = 2.5 Δε (nm) 1.13 (214) 1.22 (200) 1.44 (192)	pH = 3.5 Δε (nm) −1.36 (213) 1.81 (193)	pH = 3.7 Δε (nm) −1.44 (209) 3.51 (186)	pH = 4.2 Δε (nm) −2.86 (211)	pH = 4.4 Δε (nm) −3.04 (208)	pH = 3.7 Δε (nm) −2.38 (208) 3.72 (187)
pH = 6.3 Δε (nm) −1.27 (212)	pH = 6. 0 Δε (nm) −2.27 (210) 2.12 (195)	pH = 5.8 Δε (nm) −2.41 (212) 4.68 (193)	pH = 6.2 Δε (nm) −2.98 (210)	pH = 6.3 Δε (nm) −2.90 (208)	pH = 5.6 Δε (nm) −3.38 (211)
		pH = 7.9 Δε (nm) −0.72 (216) −1.98 (205) −1.75 (200)	pH = 8.0 Δε (nm) −3.15 (196)	pH = 8.0 Δε (nm) −1.38 (215) −2.17 (206) −2.06 (197)	pH = 8.5 Δε (nm) −0.21 (217) −1.16 (200)
pH = 9.2 Δε (nm) 2.33 (205) 2.53 (195)	pH = 9.0 Δε (nm) −0.44 (222) 0.25 (203) 0.84 (195)	pH = 9.8 Δε (nm) 1.85 (208) 1.53 (197)	pH = 10.3 Δε (nm) 0.88 (211) 1.30 (203) 0.69 (193)		

## Data Availability

All data generated or analysed during this study are included in this published article.
